# Liquid Core Waveguide Cell with In Situ Absorbance Spectroscopy and Coupled to Liquid Chromatography for Studying Light-Induced Degradation

**DOI:** 10.1021/acs.analchem.2c00886

**Published:** 2022-05-19

**Authors:** Iris Groeneveld, Ingrida Bagdonaite, Edwin Beekwilder, Freek Ariese, Govert W. Somsen, Maarten R. van Bommel

**Affiliations:** † Division of Bioanalytical Chemistry, Amsterdam Institute for Molecular and Life Sciences, Vrije Universiteit Amsterdam, De Boelelaan 1108, 1081 HZ Amsterdam, The Netherlands; ‡ Da Vinci Laboratory Solutions, Sydneystraat 5, 3047 BP Rotterdam, The Netherlands; § LaserLaB, Vrije Universiteit Amsterdam, De Boelelaan 1081, 1081 HV Amsterdam, The Netherlands; ∥ Analytical Chemistry Group, van ’t Hoff Institute for Molecular Sciences, University of Amsterdam, Science Park 904, 1098 XH Amsterdam, The Netherlands; ⊥ Conservation and Restoration of Cultural Heritage, Amsterdam School for Heritage, Memory and Material Culture, University of Amsterdam, P.O. Box 94552, 1091 GN Amsterdam, The Netherlands

## Abstract

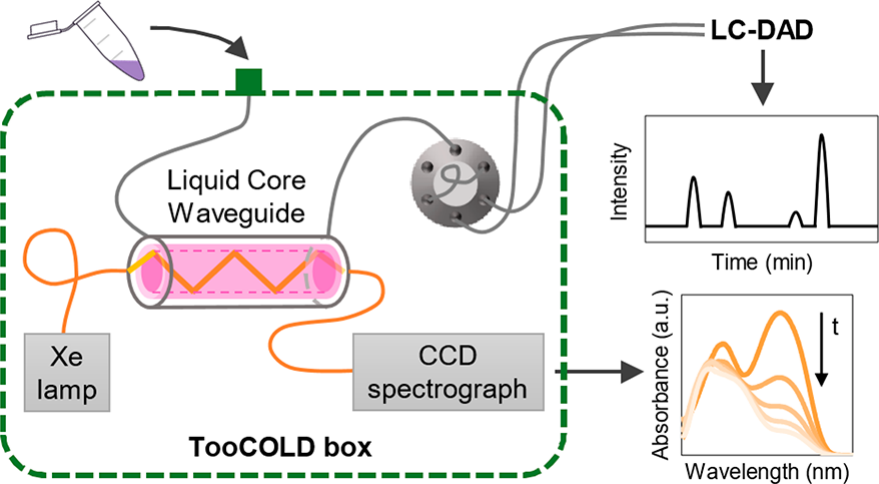

In many areas, studying photostability or the mechanism of photodegradation is of high importance. Conventional methods to do so can be rather time-consuming, laborious, and prone to experimental errors. In this paper we evaluate an integrated and fully automated system for the study of light-induced degradation, comprising a liquid handler, an irradiation source and exposure cell with dedicated optics and spectrograph, and a liquid chromatography (LC) system. A liquid core waveguide (LCW) was used as an exposure cell, allowing efficient illumination of the sample over a 12 cm path length. This cell was coupled to a spectrograph, allowing in situ absorbance monitoring of the exposed sample during irradiation. The LCW is gas-permeable, permitting diffusion of air into the cell during light exposure. This unit was coupled online to LC with diode array detection for immediate and automated analysis of the composition of the light-exposed samples. The analytical performance of the new system was established by assessing linearity, limit of detection, and repeatability of the in-cell detection, sample recovery and carryover, and overall repeatability of light-induced degradation monitoring, using riboflavin as the test compound. The applicability of the system was demonstrated by recording a photodegradation time profile of riboflavin.

## 1 Introduction

Photodegradation or light-induced degradation (LID) is the process where molecules degrade under the influence of light. There are applications where light is deliberately used to destroy molecules, for instance, for the removal of chemicals in wastewater. There are also areas where photodegradation should be prevented as much as possible, for example in art conservation, in the food and pharmaceutical industry,^1^ and for everyday products, such as wall paints, car coatings, or textile dyes. Whether it is to prevent or to actually initiate photodegradation, these processes have to be studied in a controllable and semiquantitative manner in order to achieve effective application or prevention.

Studying these photochemical processes, however, is complicated and laborious. Photodegradation is significantly influenced by a number of factors, such as the light dose, the applied wavelength, the solution pH, and the presence of other compounds or gases, such as oxygen. These parameters may affect the kinetics as well as the degradation products formed. To add to the complexity related to the many physical parameters, there is also a challenge in studying the influence that different analytes may have on each other when studying mixtures. With commonly used methods it is often difficult to find a strong link between the parent molecule and the degradation product(s). An example of such a method is where solutions are degraded in a beaker placed under a light source.^2−11^ These experiments can take rather long as the irradiation (power per area) is often low; that is, Confortin et al.^6^ irradiated a solution of crystal violet for at least 100 h, and Weyermann et al.^7^ required 54 h. Besides, errors are easily introduced since samples are taken manually, and solvents may evaporate during irradiation,^7^ resulting in irreproducible results. Hence, efficient tools are needed to study photodegradation in a simple and repeatable manner, while including the most important parameters affecting photodegradation.

In a previous report, we demonstrated the use of a liquid core waveguide (LCW) with a low refractive index (*n* = 1.29) as a LID cell with in situ absorbance detection to study photodegradation in an aqueous solution (*n* = 1.33) in an efficient way.^12^ The gas permeable LCW allows for the continuous supply of air to the photoreaction to create an environment similar to reality. In the same paper we suggested how a postseparation in online fashion could deal with the sample complexities that are met in LID studies,^12^ something that was not yet done with other photoreactors based on an LCW.^13,14^ Den Uijl et al.^15^ demonstrated that the LCW-based LID cell allows for more rapid photodegradation of crystal violet and eosin Y as compared to standard in-solution photodegradation approaches. Within the TooCOLD project (Toolbox for studying the Chemistry Of Light-induced Degradation) we have now developed the TooCOLD box, which includes the aforementioned LID cell and established coupling with liquid chromatography (LC) for analysis of the product mixture after the degradation process.

Here, we report on the analytical performance and use of this new tool using riboflavin as a model compound. Riboflavin, also known as vitamin B2, has been widely studied for its limited photostability and degradation in aqueous and organic solvents. The vitamin, which has also been applied as a textile dye,^16^ degrades rapidly under the influence of light, losing its health benefits and color characteristics. Typical degradation products include formylmethylflavin, lumichrome, lumiflavin, and carboxymethylflavin.^17−21^ Due to its very poor lightfastness, however, it has also been used as an efficient photosensitizer in order to study the photodegradation of other compounds.^22^ In short, the photodegradation of riboflavin is well-known under different circumstances; this is why it was chosen as a model compound for this study.

In this paper, we report on the linearity and linear range, limit of detection (LOD), and repeatability of the in-cell detection with and without the addition of airflow. We determined the recovery of the analyte from the LID cell for LC analysis and the repeatability of the degradation experiments. To demonstrate the applicability of the system for studying photodegradation, a time profile of the degradation of riboflavin was measured fully automatically.

## 2 Materials and Methods

### 2.1 Chemicals

For LC analysis we used ultrapure Milli-Q water (MQ), methanol (MeOH; Biosolve, UPLC/MS grade), formic acid (FA; Sigma-Aldrich), sodium hydroxide (NaOH; Sigma-Aldrich), and triethylamine (TEA; Sigma-Aldrich). All experiments were performed using solutions of riboflavin (RF; Sigma-Aldrich, pharmaceutical secondary standard) in MQ.

### 2.2 TooCOLD Setup

The TooCOLD system consists of five main parts: (i) a liquid handler, (ii) the TooCOLD box, (iii) an irradiation source, (iv) a CCD spectrometer, and (v) an LC-diode array detector (DAD) system. The complete setup is shown in Figure S1 in the Supporting Information (SI), with details of the irradiation light path in Figure S2.

#### 2.2.1 Liquid Handler

**Figure 1:**
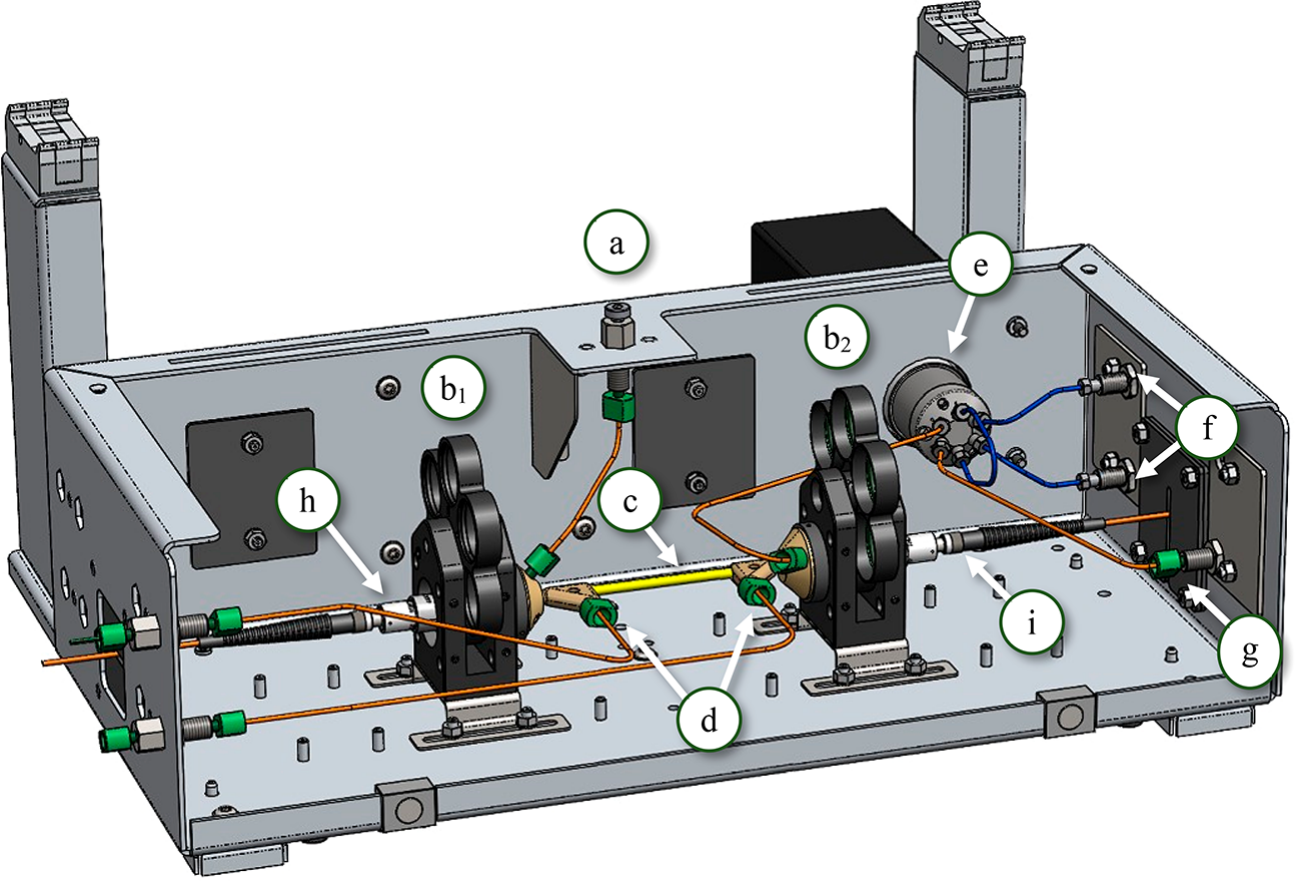
Drawing of TooCOLD box (without lid) depicting all its components: (a) injection port, (b_1,2_) filter wheels with lenses before and after the LID cell, (c) LID cell, (d) gas in- and outlet, (e) 6-port valve with 20 μL sample loop, (f) connection to LC pump and column, (g) waste channel, (h) excitation fiber with collimator, and (i) detection fiber with collimator.

The liquid handler is a Multipurpose Sampler (MPS) from GERSTEL and is controlled by MAESTRO software (GERSTEL). The MPS was configured to inject samples via the injection port of the TooCOLD box (see Figure 1a) to perform washing cycles of the LID cell and to transfer the sample from the LID cell to the LC. It was also used to trigger the CCD detector to record real-time spectra at regular intervals and the LC system to start the analysis. The methods for each experiment were designed in the MAESTRO software (see Figure S3). Injections were done with the MPS into the TooCOLD system, unless stated otherwise. In order to prevent the formation of air bubbles inside the LCW after injection, samples were always injected at a flow rate of 10 μL·s^–1^.

#### 2.2.2 TooCOLD Box

The TooCOLD box was engineered by DaVinci Laboratory Solutions based on research dedicated to the characteristics of an LCW-based LID cell which was published previously.^12^ The LID cells were fabricated by the Precision Mechanics and Engineering Group of the Vrije Universiteit Amsterdam. A schematic overview of the TooCOLD box is presented in Figure 1. The box consists of (a) an injection port, (b) two filter wheels, (c) the LID cell, which contains the LCW (Teflon AF2400, i.d., 800 μm; wall thickness, 100 μm; physical path length, 12 cm; volume, 60 μL), (d) connections for air supply (in- and outlets) to study photo-oxidation processes, (e) a 6-port valve with a 20 μL sample loop, which is connected to (f) the LC system and (g) a waste channel. The filter wheels can be used to implement optical filters to e.g. study the effect of selected wavelengths of the light spectrum or to decrease the exposure light intensity to check whether the product ratio is intensity dependent (for future experiments, not used in this study). The filter wheel holders also align the excitation fiber (h) with the LID cell, and the LID cell with the detection fiber (i).

#### 2.2.3 Irradiation Source

A 75 W Xenon short-arc fiber-coupled light source from Thorlabs (SLS205) was used for the illumination of the LID cell. The solarization-resistant excitation fiber (M112L01; i.d., 200 μm; NA = 0.22) was connected to a UV/vis collimator from Avantes (COL-UV/vis), which was attached to a plano-convex lens (Thorlabs, LA4052-ML) with a focal length of 35 mm by means of a lens tube. The collimated beam is guided through the lens tube to reach the lens so that the light was focused into the opening of the LID cell (Figure S2).

#### 2.2.4 Spectrograph

A CCD spectrometer from Thorlabs (CCS100/M) was used for real-time, in-situ monitoring of the irradiated sample inside the LCW. It was fiber-coupled to the back-end of the LID cell with the same type of lens and collimator as the excitation fiber. The spectrometer has a spectral range of 350–700 nm, with a spectral accuracy of 0.5 at 435 nm (fwhm), and a signal/noise ratio of ≤2000:1 (maximum signal/noise ratio per pixel). For light detection, it contains a 3648 pixel CCD line array, and signal integration times between 5 and 15 ms were used.

#### 2.2.5 LC-DAD

All LC analyses were carried out on an Agilent 1100 series LC system equipped with a quaternary solvent delivery system, a column oven, an autosampler, and a diode array detector (DAD). A ZORBAX Eclipse RRHD C18 column (2.1 × 150 mm; particle size, 1.8 μm) and a security guard column (2.1 × 5 mm) containing the same C18 phase were both obtained from Agilent Technologies. The LC method applied gradient elution with mobile phase A consisting of 95/5 (by volume) and B of 5/95 buffered MQ (0.1 M FA, 0.02 M NaOH, pH = 3) and MeOH, respectively, both with 5 mM TEA as an ion pairing agent. A gradient was applied at a flow rate of 120 μL·min^–1^ with first 5% mobile phase B for 1.5 min, then increasing from 5% to 95% in 15 min, followed by isocratic for 5 min. Then, the mobile phase was brought back to 5% B within 2 min, and the column was equilibrated for 5 min to be ready for the next analysis.

### 2.3 Operation of the TooCOLD System

A typical method for a 30 min degradation experiment can be found in Figure S3. In short, for each degradation experiment the MPS injected 70 μL MQ, which served as a blank sample after which it triggered the CCD spectrometer to record a reference signal (*I*_0_). Then, 70 μL of sample was injected. After this, the syringe of the liquid handler was washed with 300 μL of 75% MeOH in MQ, followed by the same volume of MQ. After washing of the needle, a transmission spectrum was recorded at *t* = 0 min for control. Then, a delay time was programmed, corresponding to the total irradiation time for the specific degradation experiment. Trigger signals to record transmission spectra were programmed at certain intervals, for example, every 10 min, to monitor the overall content of the LCW. Absorbance spectra were later calculated by the software of the CCD spectrometer. Next, the sample was transferred to the sample loop of the 6-port valve. Unless stated otherwise, this was done by injecting 50 μL of 75% MeOH in MQ to transfer the middle part of the LID cell’s content to the loop. Then, the LC was triggered by the MPS to start the analysis of the irradiated sample. Directly after the pulse was given, the 6-port valve was automatically switched from the “load” into the “inject” position, so that the sample loop was flushed with the mobile phase of the LC system. Finally, the valve was switched to the load position again and the LID cell was cleaned with 300 μL of 75% MeOH in MQ and then with pure MQ.

### 2.4 Analytical Performance

The performance of the TooCOLD setup was evaluated with regards to the in situ UV/vis spectroscopy, and repeatability of the degradation experiments. The LC method validation is presented in another report.^23^ In order to test significance, F-tests, and *t* tests were performed on the corresponding data sets.

The linearity, linear range and LOD of the in-cell absorbance measurements (optical path length, 12 cm) were determined in 5-fold. A dilution series of RF was prepared in MQ with concentrations from 1 × 10^–6^ to 13 × 10^–6^ M. Prior to each sample, a blank sample (MQ) was injected and the intensity was recorded as *I*_0_ signal at a wavelength of 450 nm.

The repeatability and stability of the in situ UV/vis detection were determined in two ways: (i) the in-cell stability by injecting a solution of RF (5 × 10^–6^ M) while measuring every 15 min for 3 h, and (ii) possible fluctuations caused by the injector by injecting the same RF solution every 15 min for 3 h and measuring the absorbance.

The TooCOLD system was also tested for recovery from the LID cell by injecting a sample of RF without subsequent irradiation and analyzing the sample by LC. The recovery was calculated by comparing the measured peak area of the RF main peak with that of a reference sample measured directly by LC-DAD while circumventing the TooCOLD box. As mentioned in section 2.3, the transfer of the sample to the loop was accomplished by addition of a so-called “transfer volume”, consisting of 75% MeOH in MQ. The tested volumes were 50, 70, or 110 μL. These volumes were chosen for specific reasons, as depicted in Figure S4: 50 μL was the physical minimum required by the MPS and allows the middle of the content of the LCW to be transferred to the sample loop; 70 μL should transfer the part of the sample present closer to the entrance of the LCW; 110 μL was chosen to determine whether the “tail” of the flushing solvent would contain significant amounts of RF. Another factor affecting the recovery was the sample loop flushing time, that is, the period of the LC mobile phase traveling through the sample loop. This flushing time was 12, 18, or 24 s, which is equivalent to 24, 36, and 48 μL when using a flow rate of 120 μL·min^–1^ for LC analysis.

The repeatability of the degradation experiments was assessed in two ways: according to the standard deviation (SD) of the decrease in absorbance of a solution of RF measured in the LCW, and the decrease in peak area of RF measured by LC after degradation.

### 2.5 Degradation Time Profile of Riboflavin

A time profile of the degradation of a solution of 5 × 10^–6^ M RF in MQ was performed in triplicate in order to test the potential of the TooCOLD system. In total, nine time points were taken: at 0 min (control, no irradiation), 30, 60, 90, 120, 150, 180, 210, and 240 min. In situ absorbance measurements of the content of the LCW were taken every 10 min. For the control measurements, a sample was injected and after 10 min without irradiation, the sample was analyzed by LC-DAD. For all other time points, a fresh sample was injected and irradiated for the designated time, followed by LC analysis. The whole experiment lasted for 54 h and was fully automated.

## 3 Results and Discussion

### 3.1 Analytical Performance

This section describes and discusses the analytical performance of the system, including the linearity, linear range, and stability of the in-cell detection with and without an active airflow surrounding the LCW. The recovery from the LID cell for LC analysis, and the repeatability of the degradation experiments were determined.

#### 3.1.1 Linearity and Linear Range

**Figure 2:**
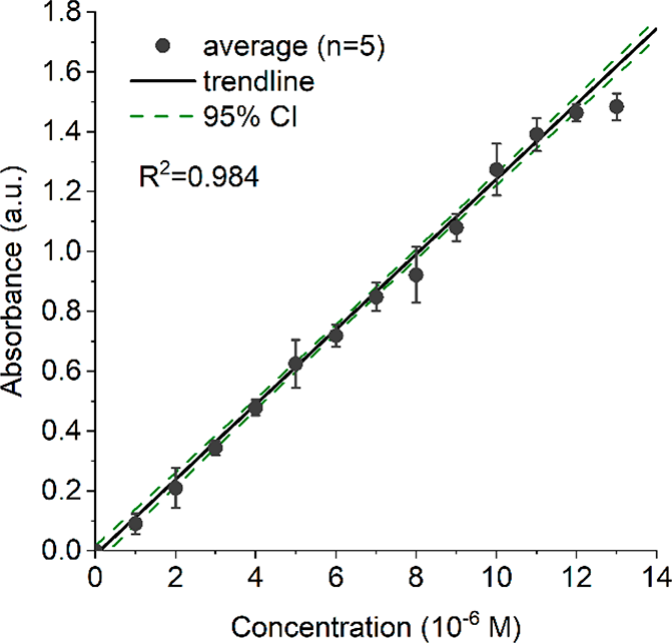
Average absorbance values with error bars of a dilution series of RF (1.0–13 × 10^–6^ M) measured in 5-fold using the LCW. A linear trendline (—) was fitted through all points and a 95% CI for the trend line was calculated (- - -).

In Figure 2 the average absorbances measured for different concentrations (1–13 × 10^–6^ M) of RF using the LCW are plotted, including the resulting linear trend line fitted through the averages. The correlation coefficient *R* was 0.984 and the relative standard deviation (RSD) ranged between 0.07 and 0.13 for the RF solutions between 3.0 × 10^–6^ and 12 × 10^–6^ M. Higher RSDs of 0.38 and 0.32 were observed for 1.0 × 10^–6^ and 2.0 × 10^–6^ M, respectively. High RSD values are to be expected for low absorbance levels since in an absorption experiment the absolute SD does not decrease for lower concentrations. The LOD for RF was calculated using the linear trend line in Figure 2 and was determined to be 1.74 × 10^–7^ M (calculation method is described in more detail in the SI). At 13 × 10^–6^ M, all measured absorbances fall outside the 95% CI of the linear fit of the absorbance–concentration relation, indicating the upper limit of the linear range (1–12 × 10^–6^ M) is reached at a measured absorbance of approximately 1.5. Therefore, for compounds absorbing in the visible range, concentrations that result in an absorbance of up to about 1.5 can be measured using this particular spectrograph. However, in order to ensure a pseudouniform analyte illumination across the whole length of the cell when studying photodegradation, the concentration of the solution and its corresponding absorbance should be considered carefully. A too high concentration may result in too strongly decreasing light intensities across the length of the cell, resulting in different degradation rates depending on the concentration and the position in the cell. This assumption was tested by irradiating solutions with different concentrations of RF for 30 min (*n* = 3) followed by LC-DAD analysis (results presented in Figure S5). As expected, higher concentrations resulted in slower degradation rates, but this effect was found to be negligible below concentrations of 6.5 μM. Therefore, we continued our study with a concentration of 5 μM, corresponding to an absorbance below 0.6 over a 12 cm long LCW, and thus lower than 0.3 for the distance to the central part of the LCW.

#### 3.1.2 System Repeatability and Stability

The repeatability and stability of the system are important features during in situ monitoring, especially because the photodegradation experiment may take up to several hours. With our setup, diffusion of air or nitrogen through the permeable LCW wall has been proven to be fairly simple to control, as demonstrated in a previous publication,^12^ and is of high interest for studying photo-oxidation reactions. The effect of applying an active airflow surrounding the LCW on the repeatability and stability was therefore evaluated.

The measurement system’s repeatability and stability were assessed in two ways: (i) injecting a single sample solution of RF (5 × 10^–6^ M) and monitoring its absorbance measurement in the LID cell over time and (ii) repeated injections and measurement of the same fresh sample solution to check for any fluctuations related to the injection system, with and without a continuous airflow around the LCW. The experiments were done without inducing LID, that is, the light source was switched on exclusively for taking a measurement every 15 min.

Table S2 shows the measured absorbances of a solution of RF in the cell over time for the four conditions. The result at 120 min for repeated injection with airflow was found to be an outlier (Grubb’s test, Table S2) and, thus, was excluded from further calculations. A relatively stable signal over a period of 3 h was observed for all conditions. The repeatability and stability were characterized by the RSD of the measurements, which for all conditions was relatively low, ranging between 0.012 and 0.024 (Table S3). To add to this, the variances were not significantly different from one another (confidence level = 95%, Table S1), proving that repeated injections and the application of a continuous airflow around the LCW do not affect the in situ absorption measurements. For further degradation experiments, a continuous airflow was applied at all times.

#### 3.1.3 Recovery

Coupling the TooCOLD box with LC may come with sample losses due to transferring the content of the LCW to LC, which should be prevented as much as possible. The transfer volume required to transfer the irradiated sample from the LCW to the sample loop was optimized (see scheme in Figure S4), together with the sample loop flushing time, that is, the time the sample loop was in the inject position. Figure S6A shows the recovery of different LCW transfer volumes, while maintaining a loop flushing time of 12 s. In Figure S6B, the results are presented when the LCW transfer volume remained constant at 50 μL, while the sample loop flushing time was 12, 18, and 24 s, respectively. The results of significance testing of the repeatability by F-tests can be found in Table S1.

It can be concluded that increasing the transfer volume from 50 to 70 μL results in a small, but significant, increase of recovery of only 3%. The error obtained for the five replicates is very low (RSD ≤ 0.003), showing that the transfer from LID cell to LC is highly repeatable. For all further degradation experiments, we applied a 50 μL volume to transfer the central part of the LCW content for LC analysis, because the first few cm of the LCW may not experience a uniform light intensity over the cross section of the LCW tube. The “tail” of the transfer solvent, obtained by using 110 μL, contained only 4% of RF, meaning that RF was efficiently removed from the LID cell. The blank runs, which were run in between each replicate of RF analysis, showed that there was no detectable carryover.

A stronger effect was observed for increasing the sample loop flushing time. A significant improvement in recovery from 73% to 87% was observed for an increase in flushing time from 12 to 18 s. With a flushing time of 24 s, the recovery further increased to 89% (Figure S6B). A flushing time of 12 s proved to be too short to completely empty the sample loop, and the lowest RSD (0.008) was obtained for a sample loop flushing time of 24 s. Based on these results, and considering the volume of the LCW (60 μL), a transfer volume of 50 μL (corresponding to the middle of the content of the LCW) and a sample loop flushing time of 24 s were used. These parameters, however, depend on the length of the tubing between the injection port, the LID cell and the 6-port valve, and the flow rate of the LC system. The transfer volume and the flushing time should, therefore, be optimized after any change to these parameters. It should also be noted that the recovery may be different for other analytes, due to the well-known “stickiness” of Teflon AF. This may be circumvented by dissolving compounds, that are otherwise easily adsorbed to Teflon, in partly organic solvent, for example, 50% methanol in water.^12^

#### 3.1.4 Degradation Repeatability

**Figure 3:**
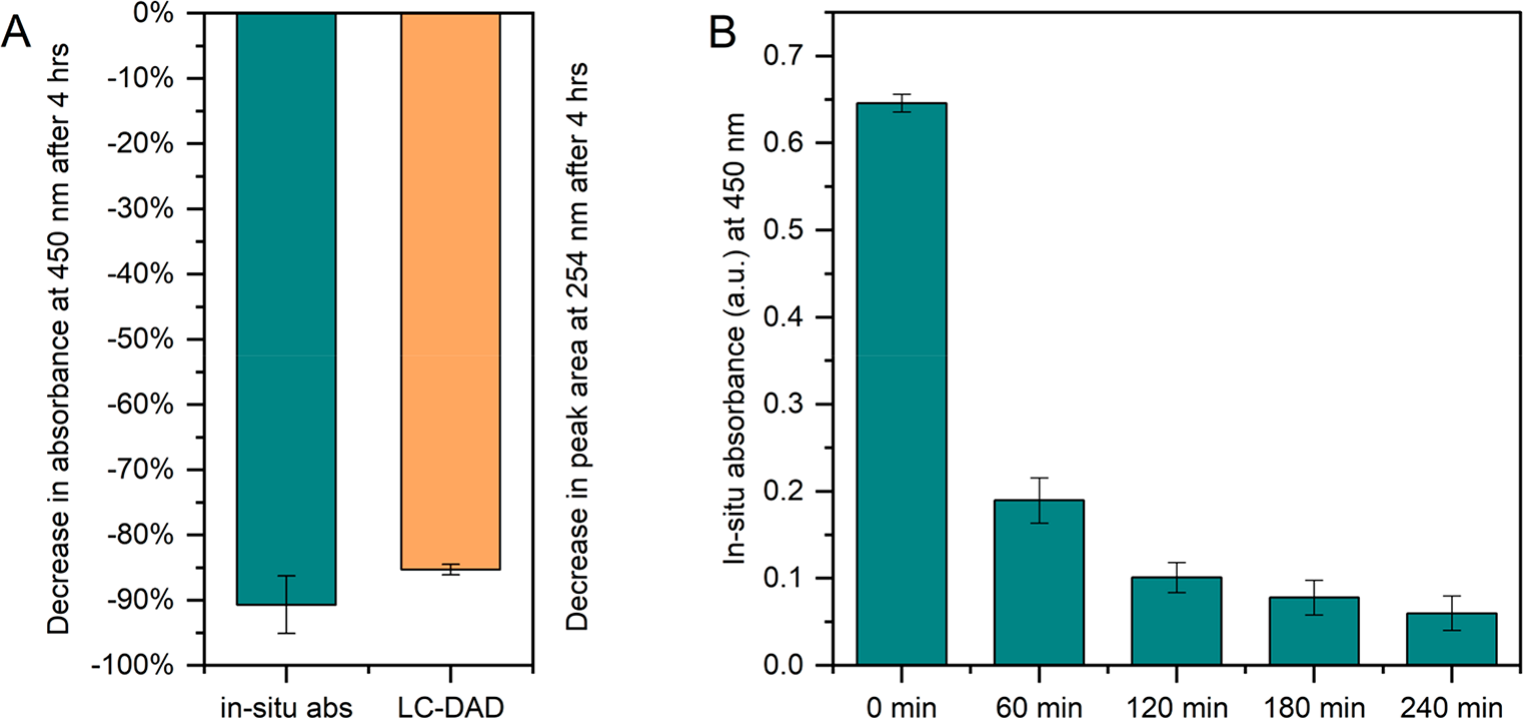
(A) Relative decrease (%) in absorbance of RF at 450 nm after 4 h of irradiation measured in situ (blue), and in peak area of RF measured by LC-DAD at 254 nm (orange). (B) Absorbances measured in situ at 60 min time intervals during the same 4 h degradation experiments of RF. Error bars indicate SD (*n* = 5).

The repeatability of the analytical system can be affected by several factors and was determined for both the in situ spectroscopic detection as well as for the degradation experiments. For a 5 × 10^–6^ M test solution of RF, which was irradiated for 4 h, Figure 3A shows the decreases of both the in situ absorbance and the peak area of RF measured with LC-DAD with error bars indicating the SD (*n* = 5). The RSDs of the peak areas of RF and degradation products are presented in Table S4 and an example of a resulting LC chromatogram can be found in Figure 5. Low RSDs of 0.038 and 0.010 were observed for in situ absorbance and peak area decreases of RF, respectively, which indicates that the TooCOLD system can monitor photodegradation in a highly repeatable manner.

The decrease in in situ absorbance (−91%) was somewhat, but significantly, larger than the decrease (−85%) observed using LC-DAD. This is rather unexpected, since the in situ measurements show the cumulative absorbance spectrum of RF and its degradation products, of which some also show absorption at 450 nm. A factor potentially contributing to the observed difference is the connecting volume between the LID cell and the six-port valve, which is about 10 μL. This may contain nondegraded RF, influencing LC analysis in case of a slight memory effect.

During the 4 h degradations the absorbance was recorded every 10 min, which is shown in Figure 3B with intervals of 60 min for legibility. With a decrease in absorbance over time, the SDs first increase, then after 1 h of irradiation remain relatively stable, as also shown in Table S5. This increase could be the result of variation introduced by the degradation process combined with the increased uncertainty associated with measuring low absorbances. Nevertheless, it was concluded that the online absorbance measurements can be used as a convenient tool to estimate the decrease of the main compound and to monitor the extent of the photodegradation process inside the LCW in real-time.

### 3.2 Degradation Time Profile of Riboflavin

A time profile of the photodegradation of RF was measured to show the applicability of the TooCOLD system. RF was irradiated in the LCW for 0 min (control, no irradiation), 30, 60, 90, 120, 150, 180, 210, and 240 min in triplicate, with a continuous airflow around the LCW, followed by LC analysis of the LCW content. The system was operated in a fully automated fashion, that is, the 2.5-day sequence was carried out unattended requiring no manual action.

**Figure 4:**
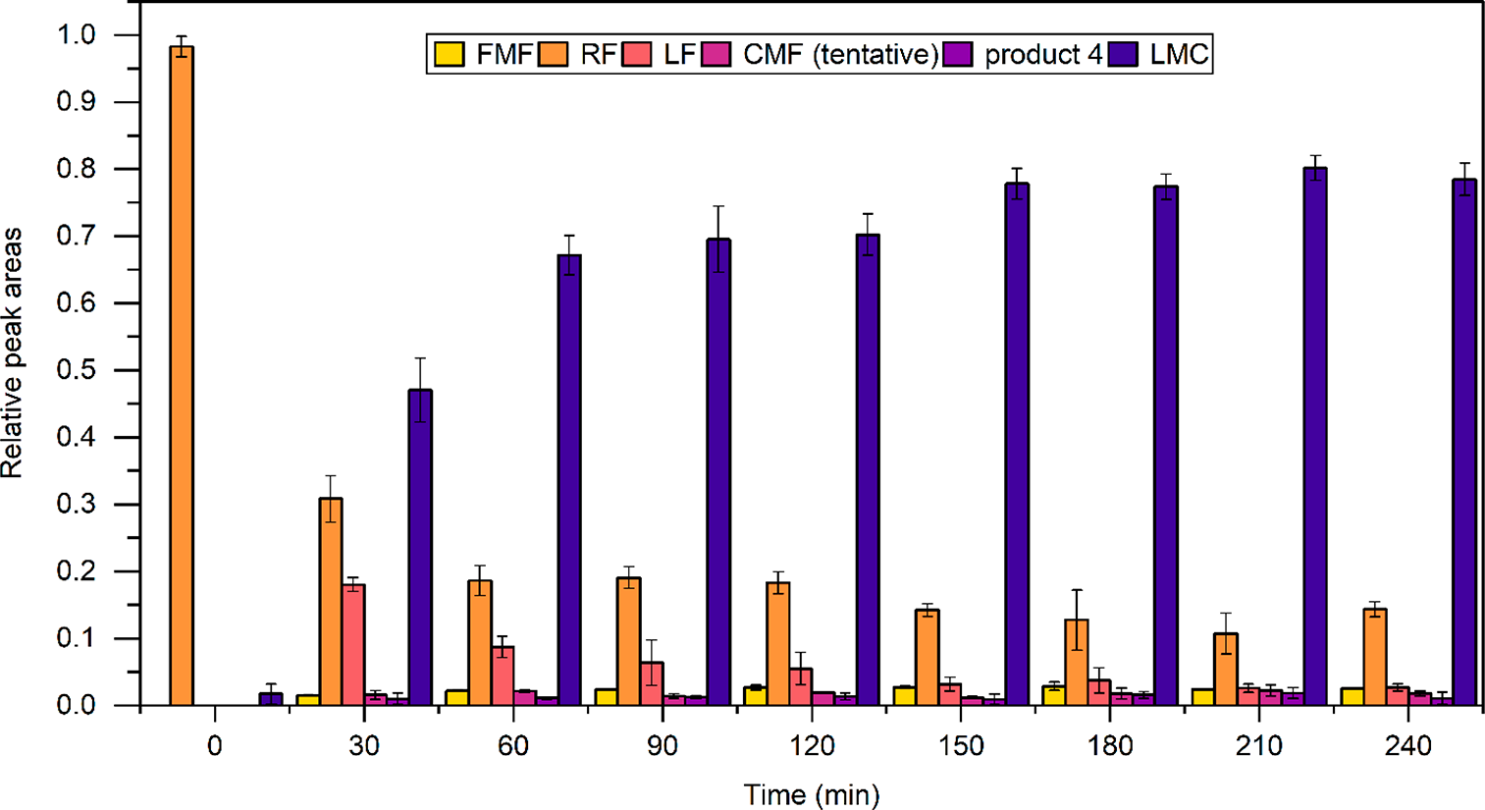
Relative peak areas of RF and five formed products measured by LC after degradation in the LCW for the indicated time; error bars indicate SDs (*n* = 3). RF was degraded for 0 (control, no irradiation), 30, 60, 90, 120, 150, 180, 210, and 240 min. Note that the LC-DAD peak areas (measured at 254 nm) will not reflect the actual relative molar concentrations, since for most products the absorption coefficients are not known and may vary.

**Figure 5:**
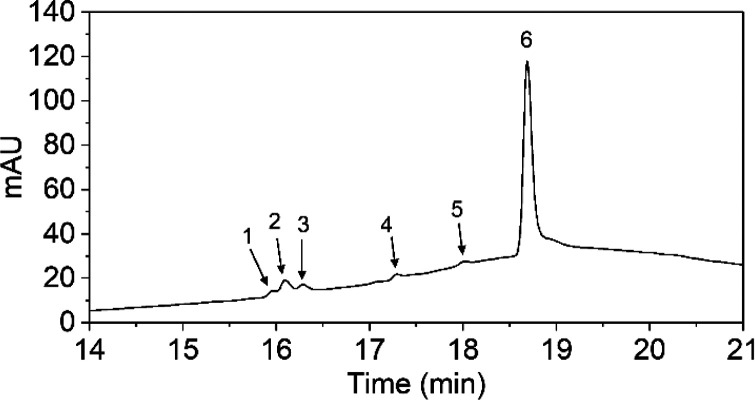
LC-DAD chromatogram recorded at 254 nm obtained after a 3 h degradation of RF. Peaks were (tentatively) identified as FMF (1), RF (2), LF (3), CMF (4), product 4 (5), and LMC (6).

**Figure 6:**
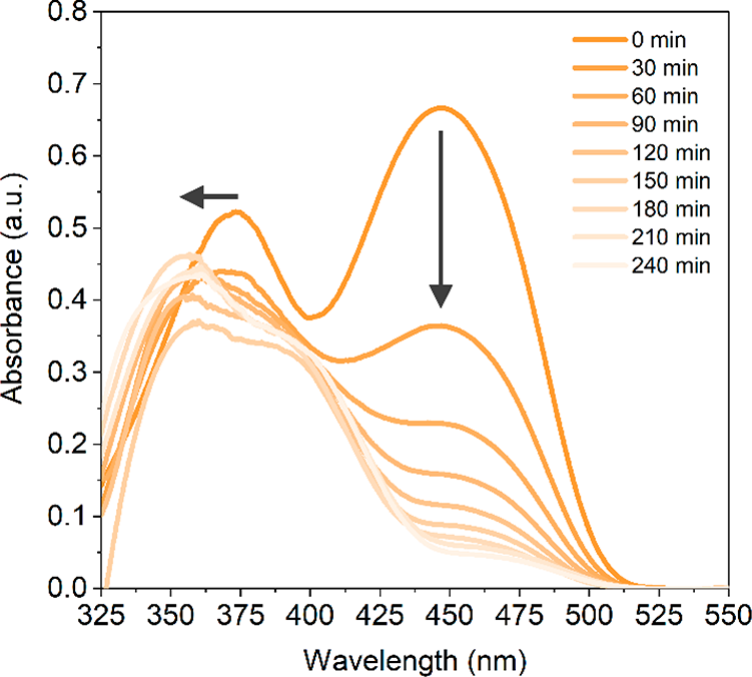
In situ absorbance spectra measured every 30 min during a 4 h degradation of RF. The vertical arrow signifies an overtime reduction in absorbance at 450 nm, and the horizontal arrow an overtime blue-shift from 375 to 350 nm, indicating a gradual change in the LCW content.

The results of the RF degradation measurements in time are presented in Figures 4, 5, and 6. Figure 4 shows the changes observed in relative peak areas measured by LC-DAD in the irradiated sample at different time points. Figure 5 shows a chromatogram of the RF solution after a 3 h degradation. The corresponding absorbance spectra recorded by the DAD are shown in Figure S7 and the analyte retention times in Table S6. Figure 6 shows the in situ absorbance spectra taken every 30 min during a 4 h degradation. Figures 4 and 6 show that the parent compound RF degraded rapidly between 0 and 30 min. After roughly 150 min, the degradation of RF seems to level off.

According to literature, the major degradation products that are formed after photolysis of RF include formylmethylflavin (FMF), lumichrome (LMC), and lumiflavin (LF) and also cyclodehydroriboflavin (CDRF) after a photoaddition pathway.^17,20,24,25^ Other minor degradation products that may be found are carboxymethylflavin (CMF) after oxidation (when oxygen is present), β-keto acid, and flavo-violet (ring-cleavage products), and 2,3-butanedione.^18^ Six distinctive peaks were found for the degraded samples when analyzed by LC (Figure 5). Five peaks that could be identified based on their retention times and absorbance spectra were FMF, RF, LF, and LMC and possibly also CMF. The sixth (product 4) had an absorbance spectrum similar to RF, but could not be identified. A few very small additional peaks were observed at 450 nm (Figure S8), but these could also not be identified based on their weak absorbance spectra. Identification of the degradation products would require coupling of the TooCOLD system to mass spectrometry, however, this was beyond the scope of the present study.

Besides the observed changes in the LC chromatograms, the changes in the in situ absorbance spectra during a 4 h degradation are shown in Figure 6. From the first measurement on, a clear change in the ratio of the absorption maxima at 350 and 450 nm (i.e., the absorption maxima of RF) can be observed. This indicates a change in composition of the sample inside the LCW, which was confirmed by LC analysis. With time, the absorption spectrum transforms into a spectrum close to that of LMC, the main degradation product. These spectra clearly show the usefulness of a spectroscopic monitoring tool coupled to the LID cell. Following the real-time change in the overall absorption spectrum may help to decide when the time has come to transfer the content of the LCW to the LC-DAD for analysis of the degradation products.

## 4 Conclusions

An integrated LCW-based LID system encompassing automated liquid handling, irradiation source and spectrograph was coupled successfully to LC-DAD and tested for its analytical performance. As demonstrated for RF, the total system allowed for quantitative unattended photodegradation experiments over longer periods of time in a highly repeatable fashion with RSDs for peak areas below 0.033. The in situ absorbance monitoring of the content of the LCW during photodegradation also showed good repeatability (RSDs of absorbance signals below 0.044). The in situ recorded absorption spectrum is the resultant of contributions from the remaining parent compound and formed degradation products. Since the spectra and absorption coefficients of the latter are often not known, the total absorbance signal should not be used for quantitative analysis. Still, it is highly useful for monitoring the progress of the degradation process in real-time. The light intensity inside the LCW can be determined using actinometry (as we demonstrated earlier^12^), but can also be approximated with a power meter behind the LCW exit. Coupling to the LC-DAD system permits separation and relative quantification of the formed degradation products based on their UV/vis response. Unambiguous identification of (unknown) degradation products and potential elucidation of reaction pathways requires the current system to be coupled to mass spectrometry, which is the objective of a follow-up study in our lab. In a future project we will study differences in photodegradation mechanisms or kinetics under aerobic versus anaerobic conditions and how results obtained in solution can help to elucidate photodegradation in solid materials where different irradiation conditions and diffusion processes play a role.

The LOD of the in situ absorbance detection is relatively low due to the optical path length of 12 cm. This characteristic comes in especially useful when degrading low analyte concentrations. Notably, at lower concentrations the LCW also ensures that the whole sample will be irradiated very efficiently and almost uniformly, resulting in faster overall photodegradation. At high analyte concentrations a large part of the incoming light may be absorbed within the first section of the LCW, and the analyte molecules in the subsequent part of the LCW will experience lower light intensities and therefore lower degradation rates.

Overall, this study demonstrates that with the TooCOLD box coupled to LC, reliable photodegradation studies can be simplified and very well automated, shortening the operator’s time spent in the lab significantly. The suitability of the system for studying relatively low analyte concentrations seems especially relevant when only very small samples are available or when an LC separation of a sample mixture is performed prior to LID. Currently, we are studying the feasibility and usefulness of a multidimensional LC-TooCOLD box-LC-DAD setup. The first outcomes of this study are very promising and we believe that integration of the LCW-based approach can transform the way photodegradation studies are performed for a wide variety of analytes.

## Supporting Information Available

Additional details about experimental methods, calculations and results, LC-DAD chromatograms and DAD recorded absorbance spectra, and a photograph of the TooCOLD-LC-DAD setup (PDF)
