# Supplementary material for: Liquid Core Waveguide Cell with In Situ Absorbance Spectroscopy and Coupled to Liquid Chromatography for Studying Light-Induced Degradation
Source: Anal Chem. 2022 May 19;94(21):7647–54. doi: 10.1021/acs.analchem.2c00886 (PMC9161219; doi:10.1021/acs.analchem.2c00886)
Supplement: Supplementary file 1 — ac2c00886_si_001.pdf [file ac2c00886_si_001.pdf]

Supporting Information

A liquid core waveguide cell with in-situ absorbance spectroscopy and coupled to liquid chromatography for studying light-induced degradation

Iris Groeneveld[1], Ingrida Bagdonaite[1], Edwin Beekwilder[2], Freek Ariese[3], Govert W. Somsen[1], Maarten R. van Bommel[4,5]

[1] Division of Bioanalytical Chemistry, Amsterdam Institute for Molecular and Life Sciences, Vrije Universiteit Amsterdam, De Boelelaan 1108, 1081 HZ Amsterdam, The Netherlands, i.groeneveld@vu.nl, +31 (0) 20 59 86 892;

[2] Da Vinci Laboratory Solutions B.V., Sydneystraat 5, 3047 BP Rotterdam, The Netherlands;

[3] LaserLaB, Vrije Universiteit Amsterdam, De Boelelaan 1081, 1081 HV Amsterdam, The Netherlands;

[4] Analytical Chemistry Group, van 't Hoff Institute for Molecular Sciences, University of Amsterdam, Science Park 904, 1098 XH Amsterdam, The Netherlands;

[5] Conservation and Restoration of Cultural Heritage, Amsterdam School for Heritage, Memory and Material Culture, University of Amsterdam, P.O. Box 94552, 1091 GN, Amsterdam, The Netherlands.

Table of contents

The TooCOLD-LC-DAD set-up	p. S3
A typical method programmed in Maestro for a degradation experiment	p. S5
Schematic overview of transfer volumes tested	p. S6
Effect of riboflavin concentration on degradation rate	p. S7
Overview of significance testing by F- and t-tests	p. S8
Calculation of LOD for riboflavin from in situ absorbance measurements	p. S10
Data obtained from spectral repeatability experiments	p. S11
Data for recovery experiments	p. S12
Additional data for repeatability of analysis and degradation	p. S13
Additional data from LC analysis of riboflavin time profile	p. S14

The TooCOLD-LC-DAD set-up

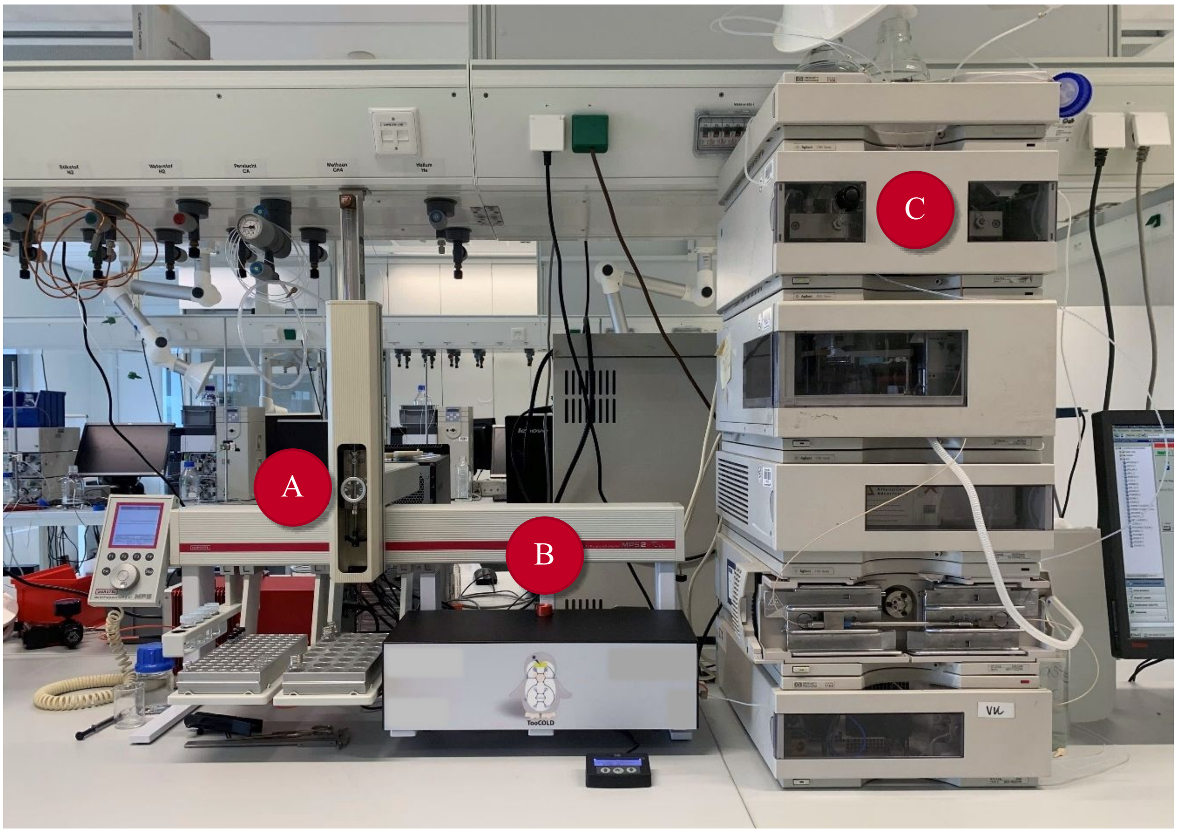

Figure S1. Photo of the TooCOLD system with (A) the liquid handler, (B) the TooCOLD box, and (C) the LC-DAD system.

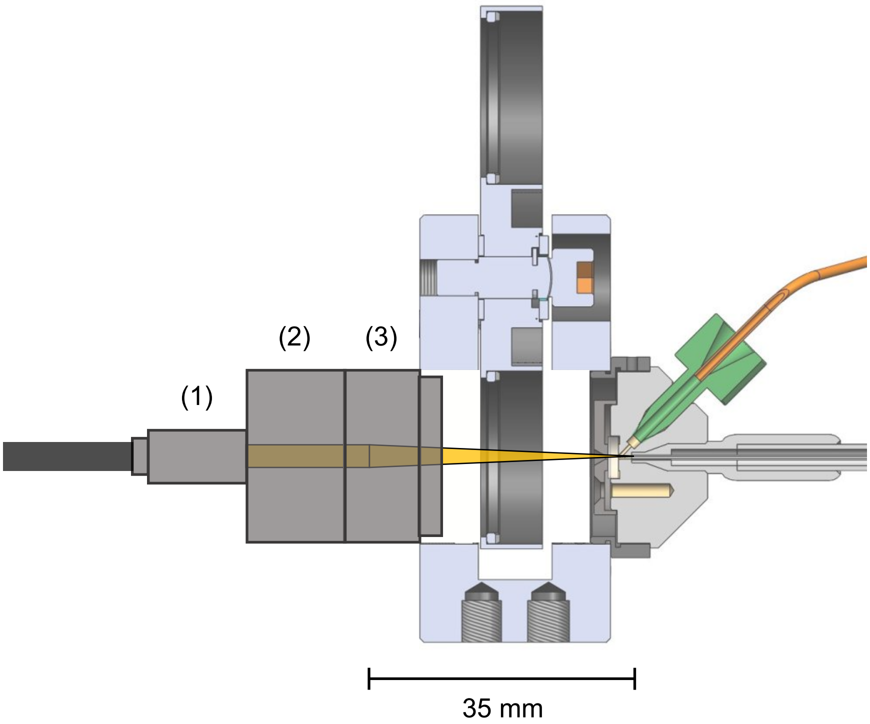

Figure S2. A cross section of the filter wheel holding the optical elements on the left and the LID cell on the right. From left to right: a collimated beam (yellow) leaves the collimator (1), that is connected to a lens (3) via a short lens tube (2). The collimated beam is then focused by the lens, which has a focal length of 35 mm so that the focal point is positioned closely to the entrance of the LCW.

A typical method programmed in Maestro for a degradation experiment

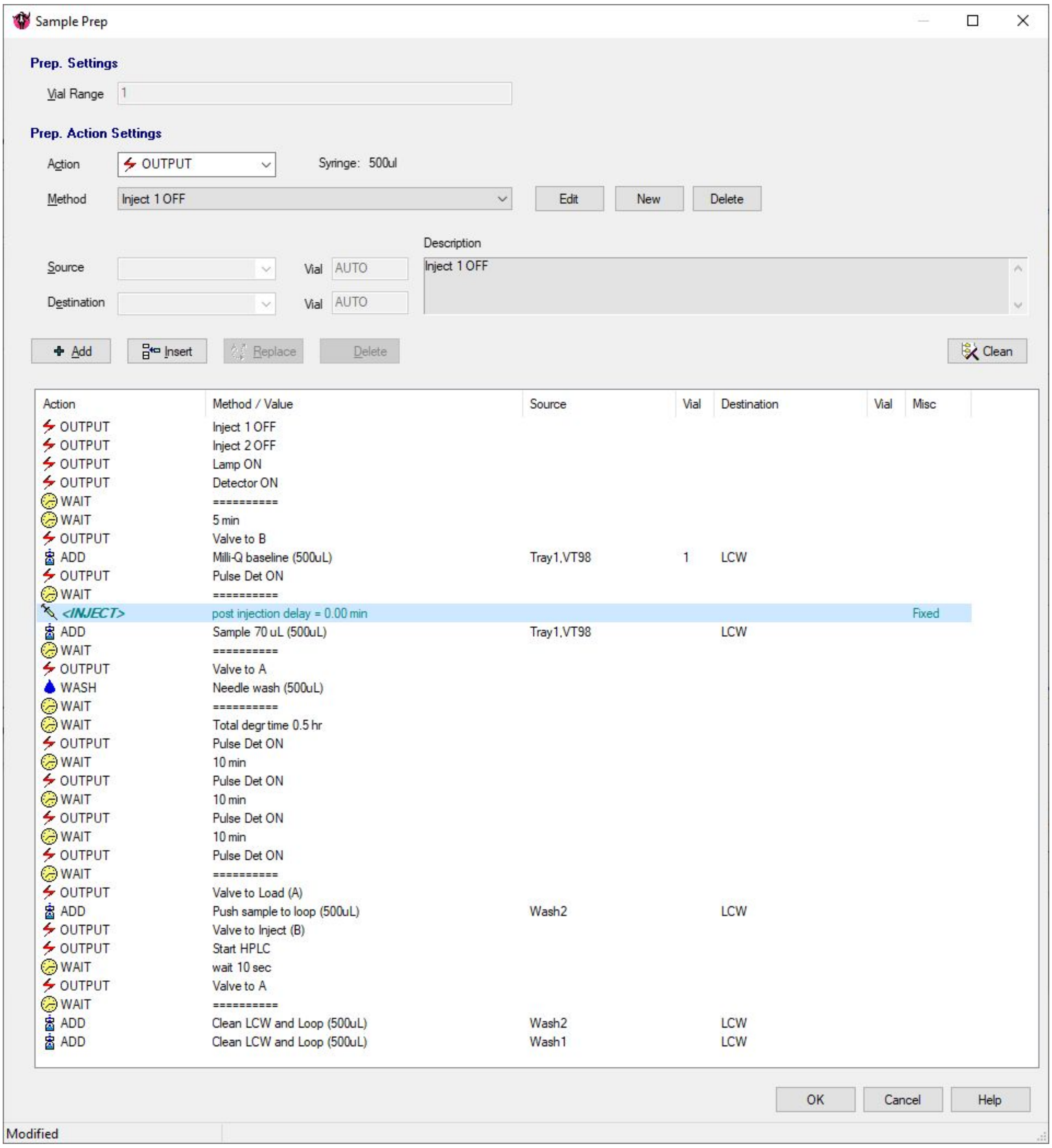

Figure S3. An overview of a typical method programmed in Maestro to perform all steps during a 30 min degradation experiment. The first steps indicate that the lamp and detector are switched on. The 6-port valve is switched to position B (inject position). A sample of Milli-Q is injected to measure the baseline (I_0). 'Pulse Det ON' sends a trigger to the spectrometer to measure an absorbance spectrum. The command 'Sample 70 µL' injects a sample from the sample tray into the LID cell. The needle is then washed and real-time absorbance spectra are taken every 10 min. Then, the valve is switched to A (load) to transfer the sample to the sample loop. It is then injected and measured by LC. Afterwards, the LID cell and sample loop are flushed with wash solvent 2, followed by wash solvent 1.

Schematic overview of transfer volumes tested

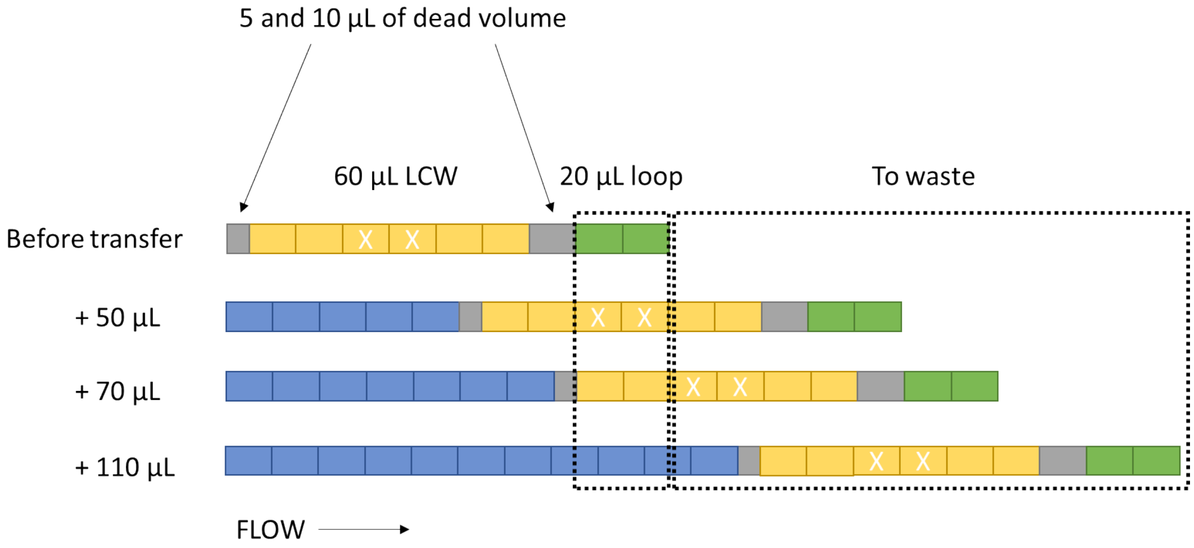

Figure S4. A schematic overview of the transfer volumes tested to optimize the recovery of RF measured by LC; each rectangle represents 10 µL. The total volume of the LCW is 60 µL, the sample loop is 20 µL and there is a total of 15 µL of tubing volume that connects the injection port with the LID cell and the LID cell with the 6-port valve. The sample loop is depicted in green, the transfer volume in blue, the volume of the LCW in yellow. The middle of the cell is indicated with two X's. In the most ideal scenario the middle of the cell is transferred to the sample loop and the rest is sent to the waste channel.

Effect of riboflavin concentration on degradation rate

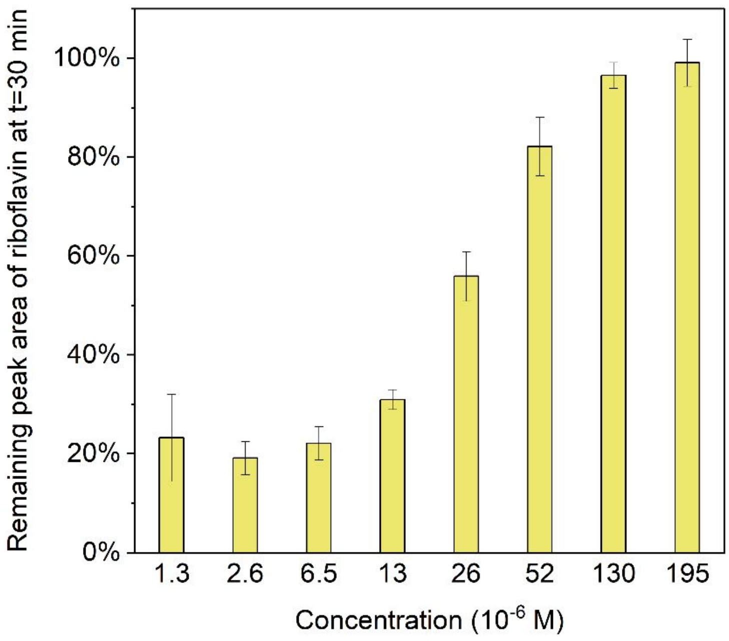

Figure S5. The remaining peak area of riboflavin measured by LC-DAD after 30 min of irradiation for different concentrations (n=3) compared to the peak area before irradiation. The degradation rate is dependent on the concentration, with a decreasing rate at higher RF concentrations. This is due to high absorbance at the front of the LCW, resulting in low light intensities reaching the middle section of the LCW (from where the solution will later be transferred for LC analysis). This effect becomes negligible at concentrations below $6.5*10^{-6}$ M RF.

Overview of significance testing by F- and t-tests

Table S1. Each hypothesis was tested by applying a two sided t-test. Beforehand, a two-sided F-test was performed to conclude if a t-test for equal or unequal variances had to be applied. n=number of replicates, x=average, s=standard deviation. Critical values for F and t at a confidence level of 95% are shown in black, the calculated values for F and t are expressed in green if H0 can be accepted, or in red when H0 is false. The number of replicates are different for question 2, which is because of removal of an outlier.

Question		Hypothesis	Test	n1	n2	x1	x2	s1	s2	$F_{critical, 2\text{-}sided}$	$F_{calculated}$	$T_{critical, 2\text{-}sided}$	$T_{calculated}$
1. Is there a significant difference in repeatability with or without an airflow for in-situ absorbance measurements?	**H0**<br>H1	**There is no difference**<br>There is a difference	F-test	13	13	0.712	0.726	0.009	0.015	**3.277**	2.897		
2. Is there a significant difference in repeatability with or without an airflow for repeated injection?	**H0**<br>H1	**There is no difference**<br>There is a difference	F-test	13	12	0.628	0.720	0.015	0.015	**3.277**	1.095		
4. Is there a significant difference in relative recovery of RF between a transfer volume of 50 and 70 uL?	H0<br>**H1**	There is no difference<br>**There is a difference**	F-test and t-test	3	3	425.5	442.9	1.114	1.595	**39.00**	2.051	**2.776**	-15.52
5. Is there a significant difference in relative recovery of RF between a transfer volume of 70 and 110 uL?	H0<br>**H1**	There is no difference<br>**There is a difference**	F-test and t-test	3	3	442.9	19.17	1.595	1.115	**39.00**	2.046	**2.776**	377.2
6. Is there a significant difference in relative recovery of RF between a transfer volume of 50 and 110 uL?	H0<br>**H1**	There is no difference<br>**There is a difference**	F-test and t-test	3	3	425.5	19.17	1.114	1.115	**39.00**	1.003	**2.776**	446.6
7. Is there a significant difference in repeatability between a flushing time of 12 and 18 s?	**H0**<br>H1	**There is no difference**<br>There is a difference	F-test	5	5	721.4	853.9	33.55	35.30	**9.605**	1.107		

**Question**		**Hypothesis**	**Test**	**n1**	**n2**	**x1**	**x2**	**s1**	**s2**	**$F_{critical, 2\text{-}sided}$**	**$F_{calcula\text{-}ted}$**	**$T_{critical, 2\text{-}sided}$**	**$T_{calcula\text{-}ted}$**
8. Is there a significant difference in repeatability between a flushing time of 18 and 24 s?	H0<br>**H1**	There is no difference<br>**There is a difference**	F-test	5	5	853.9	869.9	35.30	7.326	**9.605**	**23.216**		
9. Is there a significant difference in repeatability between a flushing time of 12 and 24 s?	H0<br>**H1**	There is no difference<br>**There is a difference**	F-test	5	5	721.4	869.9	33.55	7.326	**9.605**	**20.970**		

Calculation of LOD for riboflavin from in situ absorbance measurements

The LOD of riboflavin (on-line absorbance measurements in the 12-cm LCW) was calculated by means of the linear trend line, shown in Section 3.1.1. The LOD is determined as the concentration where the signal is three times the SD of the noise (σ_{noise}). The value for σ_{noise} was obtained from the baseline of a spectrum of a blank measurement.

Formula of the linear trendline in Figure 2:
$y = 122012.42x - 0.0124$

$\sigma_{noise} = 0.0086$

$3^* \sigma_{noise} = 0.0168$

$0.0168 = 122012.42x - 0.0124$

$\boldsymbol{x = 1.74^*10^{-7}}$ **M**

Data obtained from system repeatability experiments

Table S2. Absorbances measured in-situ obtained from spectral stability measurements with the possible outlier highlighted in red.

	Time (min)												
	0	15	30	45	60	75	90	105	120	135	150	165	180
Inside LCW	0.702	0.711	0.700	0.717	0.704	0.714	0.718	0.702	0.718	0.721	0.705	0.719	0.726
Repeated injection	0.598	0.624	0.638	0.636	0.646	0.650	0.617	0.614	0.633	0.615	0.638	0.614	0.643
Inside LCW + airflow	0.758	0.746	0.726	0.726	0.734	0.716	0.726	0.723	0.720	0.708	0.709	0.731	0.711
Repeated injection + airflow	0.712	0.719	0.730	0.728	0.730	0.727	0.733	0.704	0.583	0.719	0.737	0.688	0.705

Grubb's test:

$G_{critical}$ with a confidence level of 95% for 13 tests is 2.29

Suspected outlier = 0.583

Average 'repeated injection + airflow' = 0.709

SD 'repeated injection + airflow' = 0.040

$G_{calculated}$ = |(outlier – average)|/SD = |(0.583 – 0.709)|/0.040 = 3.118

$G_{calculated}$ is larger than $G_{critical}$, and the value is therefore identified as an outlier and was excluded from the dataset. An overview of the new averages and RSDs are shown in Table S3.

Table S3. Average absorbances and RSDs after removal of the detected outlier.

**Outlier removed**	**Average absorbance**	**SD**	**RSD**
Inside LCW	0.71	0.0086	0.012
Repeated injection	0.63	0.0154	0.024
Inside LCW + airflow	0.73	0.0146	0.020
Repeated injection + airflow	0.72	0.0147	0.020

Data for recovery experiments

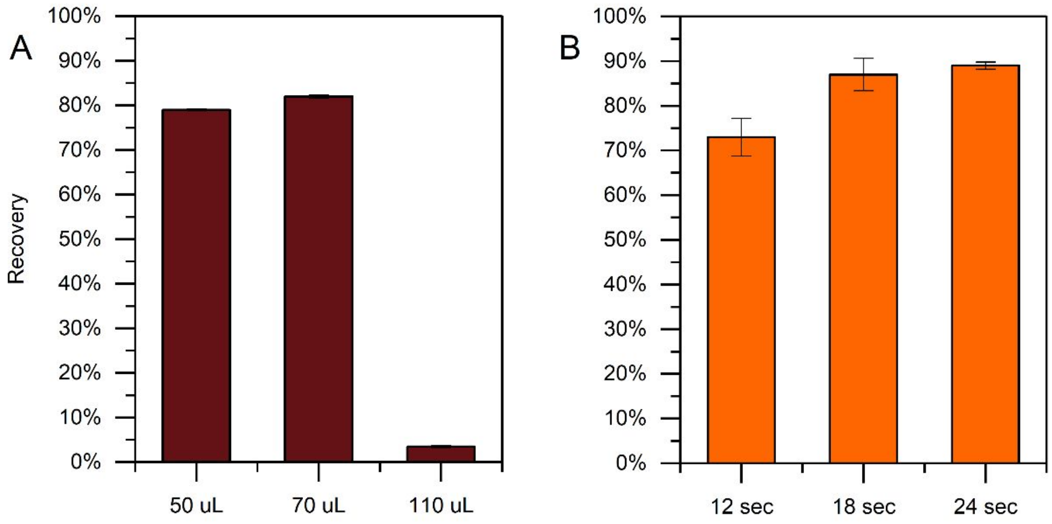

Figure S6. A: recovery of RF from the LID cell obtained with transfer volumes of 50, 70 and 110 μL measured by LC. B: recovery of RF from the LID cell obtained with loop flushing times of 12, 18 and 24 s measured by LC.

Additional data for repeatability of analysis and degradation

Table S4. Overview of the RSDs of relative peak areas after 4-hr degradations of RF measured by LC.

**Relative standard deviations of peak area of RF and degradation products**					
	P1	RF	P3	P4	P5
**RSD**	0.002	0.010	0.004	-	0.015

Table S5. Overview of the average absorbances measured in-situ, SDs and RSDs at different time points during 4-hr RF degradations in five-fold.

	**0 min**	**60 min**	**120 min**	**180 min**	**240 min**
**average absorbance (n=5)**	0.646	0.190	0.101	0.078	0.060
**SD**	0.010	0.026	0.017	0.020	0.020
**RSD**	0.015	0.137	0.172	0.254	0.331

Additional data from LC analysis of riboflavin time profile

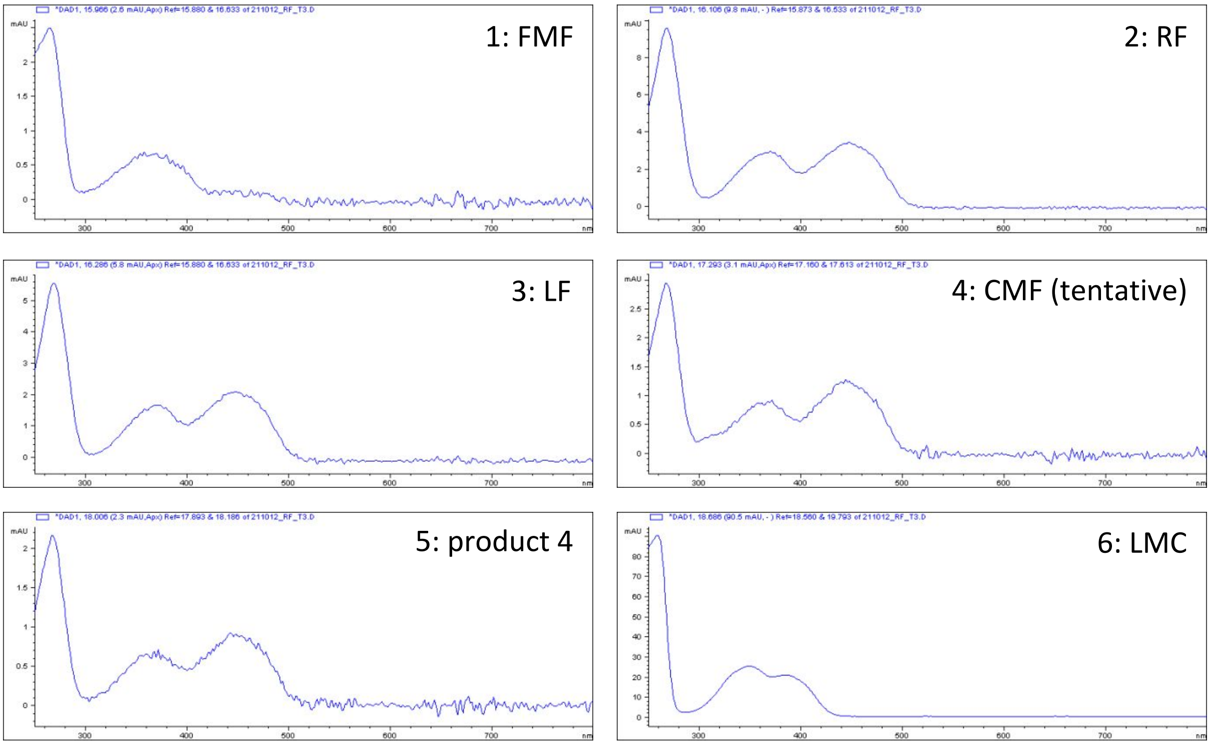

Figure S7. LC-DAD spectra of the reaction products obtained after 3-hr degradation of RF, see Fig. 5 for the chromatogram. The six major peaks were (tentatively) identified according to their absorbance spectra: 1=FMF; 2=RF; 3=LF; 4=tentatively identified as CMF; 5=product 4, could not be identified; 6=LMC.

Table S6. An overview of the six major peaks recorded at 254 nm in the LC chromatogram, stating the retention times and absorbance maxima of the identified compounds.

#	Retention time (min)	Absorbance maxima (nm)	Identified compound
1	15.96	265, 365	FMF
2	16.09	268, 370, 448	RF
3	16.28	269, 370, 446	LF
4	17.29	269, 370, 446	CMF (tentative)
5	18.00	266, 370, 448	'Product 4'
6	18.69	259, 350, 385	LMC

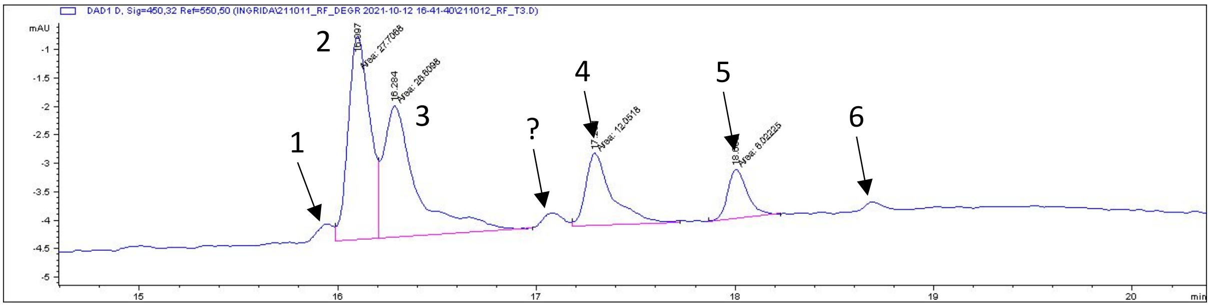

Figure S8. LC chromatogram of the same sample as in Figure 5 but DAD absorbance extracted at 450 nm, where a 7th peak (indicated with ?) was observed.
